# Investigation of Strain Effects on Photoelectrochemical Performance of Flexible ZnO Electrodes

**DOI:** 10.1038/s41598-019-47546-1

**Published:** 2019-07-29

**Authors:** Nazrin Abdullayeva, Cigdem Tuc Altaf, Merve Mintas, Ahmet Ozer, Mehmet Sankir, Hamza Kurt, Nurdan Demirci Sankir

**Affiliations:** 1 Micro and Nanotechnology Graduate Program, TOBB University of Economics and Technology, Sogutozu Caddesi No 43 Sogutozu, 06560 Ankara, Turkey; 2 Department of Materials Science and Nanotechnology Engineering, TOBB University of Economics and Technology, Sogutozu Caddesi No 43 Sogutozu, 06560 Ankara, Turkey; 3 Department of Electrical and Electronics Engineering, TOBB University of Economics and Technology, Sogutozu Caddesi No 43 Sogutozu, 06560 Ankara, Turkey

**Keywords:** Materials science, Engineering

## Abstract

In this report, the growth of zinc oxide (ZnO) nanocrystals with various morphologies, nanoflower, nanosheet, and nanorod, on flexible stainless steel (SS) foils to be utilized as photoanodes in photoelectrochemical (PEC) solar cells has been presented. It has been aimed to provide flexibility and adaptability for the next generation systems with the incorporation of SS foils as electrode into PEC cells. Therefore, physical deformation tests have been applied to the prepared ZnO thin film photoanodes. These thin films have been thoroughly characterized before and after straining for better understanding the relationship between the morphology, straining effect and photoelectrochemical efficiency. We observed a notable increase in the maximum incident photon-to-current efficiency (IPCE) and durability of all ZnO photoelectrodes after straining process. The increase in IPCE values by 1.5 and 2.5 folds at 370 nm has been observed for nanoflower and nanorod morphologies, respectively after being strained. The maximum IPCE of 69% has been calculated for the ZnO nanorod structures after straining. Bending of the SS electrodes resulted in the more oriented nanorod arrays compared to its flat counterpart, which improved both the light absorption and also the photo-conversion efficiency drastically. The finite-difference time-domain simulations have also been carried out to examine the optical properties of flat and bent ZnO electrodes. Finally, it has been concluded that SS photoanodes bearing ZnO semiconducting material with nanoflower and nanorod morphologies are very promising candidates for the solar hydrogen generator systems in terms of efficiency, durability, flexibility, and lightness in weight.

## Introduction

A tremendous number of researches have been conducted on renewable energy sources in the last decades^1–3^. Continuous investigations performed in this field have shown that solar energy is undoubtedly the most abundant and powerful source of energy, being an important alternative to commonly used fossil fuels such as coal, oil and natural gas. Particularly, the generation of solar hydrogen via photoelectrochemical (PEC) water splitting plays an important role in the field of renewable energy and is a highly promising technique for future energy conversion applications^4–6^. Since the first introduction of TiO_2_ photoelectrode into solar-driven water splitting by Honda and Fujishima^7^, the numerous researches have been reported the utilization of different semiconductor oxides such as iron oxide (Fe_2_O_3_), titanium dioxide (TiO_2_) and zinc oxide (ZnO) as photoanode materials^8–12^. Among these metal oxides, ZnO perfectly matches with the desired properties of optoelectronics due to its higher electron mobility compared to the other metal oxides, excellent stability, and large exciton binding energy (60 meV)^13,14^. Hence, it has been used in several optoelectronic application areas including light-emitting diodes, panel displays, sensors^15^, photodetectors^16^, piezoelectrical applications^17^, and solar cells^18^. A variety of synthesis techniques have been employed for the formation of ZnO nanostructures^19–22^. Among all, chemical bath deposition (CBD) is one of the simplest yet effective synthesis methods developed so far. It is a low energy requiring process since the growth of desired ZnO nanostructures is maintained inside an aqueous solution with a zinc precursor at temperatures below 100 °C. Three dimensional (3D) ZnO nanostructures with the high surface area are more advantageous since they surge the electron-hole pair generation and the absorptivity of ZnO films in the visible range. Several studies investigating the optical, electrical and photoelectrochemical properties of 1D/3D ZnO films have been handling the nanowire^23^, nanorod^24^, nanoflower^25^, nanourchin^26^, nanopetal^27^ and other structures prepared by various methods reported in the literature. In our recent study, the PEC performances of various ZnO morphologies growing on fluorine doped tin oxide coated glass have been investigated, as well^28^.

The growing demand for flexible electronic devices pushes forward the utilization of polymer or metal foils and meshes as substrates in thin film solar cell applications^29–32^. A special interest has been paid to stainless steel (SS) meshes due to their unique geometry, light-weight, and flexibility^33–36^. Li *et al*. reported the growth of porous nanosheet-based hierarchical ZnO on compacted SS meshes (CSSMs) for dye-sensitized solar cell applications. CSSMs compared to standard meshes have been considered to be more efficient due to their large surface area, and porosity serving for easy light absorption compared to the fully dense and flat substrates^34^. Another study based on ZnO growth on SS mesh has been reported by Hsu *et al*., where the Ag_2_S-coupled ZnO@ZnS core-shell nanorods are called as 3D heterostructures for photocatalytic hydrogen generation performances. The highest generated photocurrent of 60 µA has been observed for ZnO-ZnS core-shell structures^35^. In another study, Ong *et al*. have reported a solution processable growth of ZnO nanowire heterostructured arrays loaded with platinum, silver and copper oxide on SS mesh for photodegradation of methyl orange dye^36^. It is possible to conclude that SS meshes show a good performance if 1D nanocrystals, such as nanorods and nanowires, are deposited on the surface. However, it is very hard to deposit continuous and well-adhered 3D-ZnO nanostructures, such as nanoflowers and nanosheets on the SS mesh due to the micron-size spherical features of these morphologies. Hence, there is a strong need for the investigation of 3D-ZnO nanostructures on flexible substrates for PEC applications. Although numerous studies have been reported on the growth of ZnO on several types of metal foils, such as zinc or titanium, there are still very limited numbers of works reported in literature focused on the ZnO deposition on SS foils, especially for solar driven hydrogen generation systems^29–31,37,38^. SS foil offers the cost advantage compared to the other metal foils. Besides, unlike the mesh structure, SS foil allows the complex 3D-ZnO nanocrystal growth on the surface. Moreover, there is a need for better understanding of the effects of the straining on the optoelectronic properties of ZnO based photoelectrodes deposited on flexible substrates like SS foil. Therefore, in this study, we have primarily focused on the chemical bath deposition of the 1D- and 3D-ZnO nanostructures on SS foils. In addition to morphological and optical material characterizations, the incident photon-to-current efficiencies (IPCE) and PEC performances of hydrothermally deposited ZnO thin films have been investigated. IPCE values that represent the ratio between the incident photons on the active site of material and the total number of collected carriers have been measured via monochromatic light generator at different wavelengths ranging between 367–550 nm. The performance values have been compared in order to detect the changes after straining. The theoretical background behind the changes in IPCE values and light absorption properties has been studied via “Lumerical FDTD Simulation Software”^39^. These numerical integration approaches have shed light on the variations in the optical properties of ZnO nanostructures after straining. According to our best knowledge, there is no previous work reporting the straining effect on the morphological, optical and photoelectrochemical properties of 1D/3D ZnO nanostructures deposited on stainless steel foils supported with FDTD computational studies. As a conclusion, this study is going to enlighten the path towards the utilization of curvature designed thin film solar cell systems for future energy conversion applications. The rest of the paper is organized as follows: We first provide properties of flat and strained ZnO electrodes in Section 2.1 and 2.2, respectively. Then, Electrochemical Impedance Spectroscopy (EIS) measurements of different ZnO nanostructured thin films are presented in Section 2.3. Numerical investigation of light absorption properties of nanoflowers and nanorods is given in Section 2.4. The results corresponding to the cyclic deformation of ZnO electrodes are shared in Section 2.5. Finally, Section 3 summarizes our key findings.

## Results and Discussion

### Properties of flat ZnO electrodes

**Figure 1:**
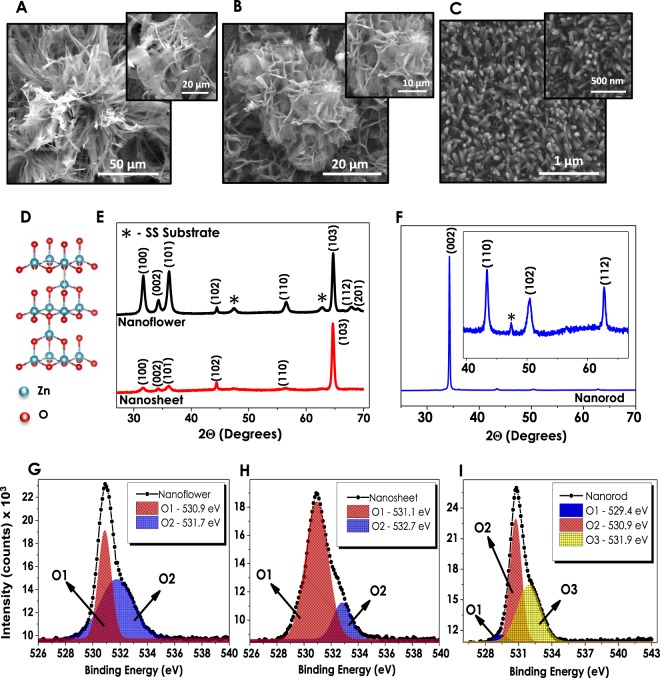
Scanning electron microscopy images of (**A**) nanoflower, (**B**) nanosheet, and (**C**) nanorod structures; (**D**) representation of wurtzite crystal structure and (**E**–**F**) XRD data of NF, NS and NR structures separately; (**G**–**I**) high resolution  O1s XPS spectra deconvoluted peaks of NF, NS and NR samples, respectively.

The effect of a change in anionic species on the formation of ZnO nanostructures has been investigated via SEM analysis. Figure 1A shows the SEM images of 3D nanostructures of ZnO deposited using Zn(C_4_H_6_O_4_.2H_2_O). Deploying zinc acetate based anion source in the solution resulted in the nanoflowers (NF) resembling chive flowers with approximately 100 μm diameters possessing distorted petal-like formations in it. On the other hand, zinc nitrate hexahydrate precursor, forming a less dense film on the same substrate, gives a nanosheet-like (NS) composition (Fig. 1B). Although the adhesion of NS film on the surface is good enough, the uniformity of the overall structure is less dense (Fig. S1, Supplementary Information). Moreover, the sizes of NS structures are smaller than that of NFs, which is related to the different nucleation and growth mechanisms of the given 3D structures^28^. The ZnO nanorod (NR) formation is observed as highly dense and uniform, possessing the structure with the average diameter and length of ~100 nm and ~1.4 μm, respectively (Figs 1C & S2). The favorable surface adhesion properties of all three structures have been confirmed via standard scotch-tape test. Table 1 displays the elemental composition data obtained from Energy Dispersive X-Ray Spectroscopy Analysis (EDAX) of ZnO thin films (Fig. S3). Among all three structures, NF is the one containing the highest atomic percentage of Zn, while NR possesses the lowest amount. The given data indicate that NF contains oxygen vacancies (Vo), whereas NR has zinc vacancies. The SEM images of the bare SS foil and ZnO seeding layer deposited SS foil are given in Fig. S4 for comparison.

**Table 1:** Zn:O Ratios for ZnO Nanostructures Obtained from EDAX Analysis.

ZnO Thin Film Morphologies	Elemental composition (at. %)		Zn/O Ratio
	Zn	O	
Nanoflower	58.56	38.76	1.5/1.0
Nanosheet	38.25	39.66	1.0/1.0
Nanorod	12.88	19.34	1.0/1.5

XRD analysis has been conducted for understanding the crystallinity and composition of synthesized ZnO nanostructures (Fig. 1E–F). The phase and crystal structure can be identified according to the XRD patterns obtained from 3 different ZnO morphologies. The obtained patterns signify the wurtzite hexagonal structure (Fig. 1D) formation with Miller’s indexes at (100), (002) and (101) crystallographic planes for all 3D morphologies^34^. In accordance with SEM images, the obtained ZnO NF structure has higher peak intensities in its diffraction pattern, which in comparison to NSs can be related to better crystallinity and higher film thickness^35^. On the other hand, XRD patterns of NRs showed the strongest diffraction peak at (002) crystal plane which stands for the preferred film orientation in c-axis. The dominance of (002) peak indicates that NRs have a highly preferred growth in the direction which is perpendicular to the substrates^36^. The fact that peaks at (102), (110) and (112) are unnoticeably small compared to the one at (002) plane suggests that the growth rate of NR films in other orientations is much slower.

**Figure 2:**
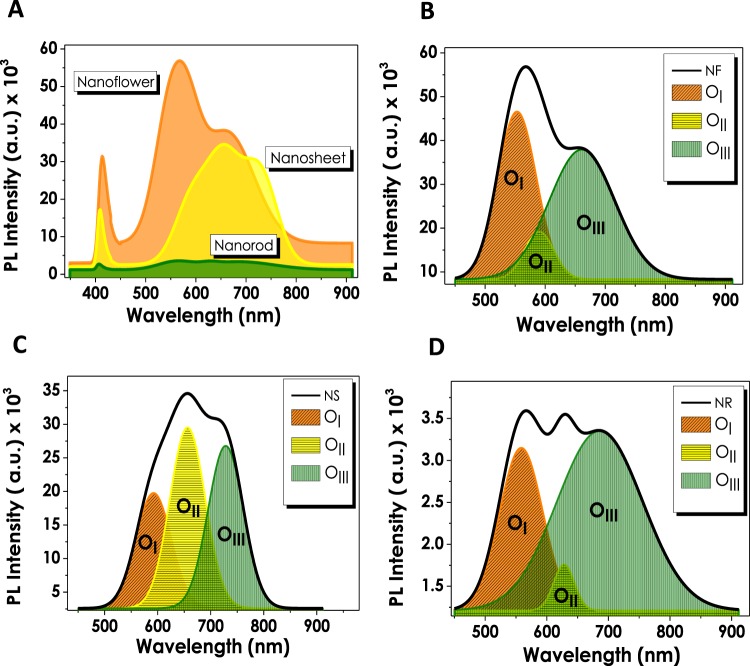
(**A**) Photoluminescence spectra of ZnO nanostructures and individual PL deconvolutions of (**B**) nanoflower, (**C**) nanosheet, and (**D**) nanorod morphologies.

**Figure 3:**
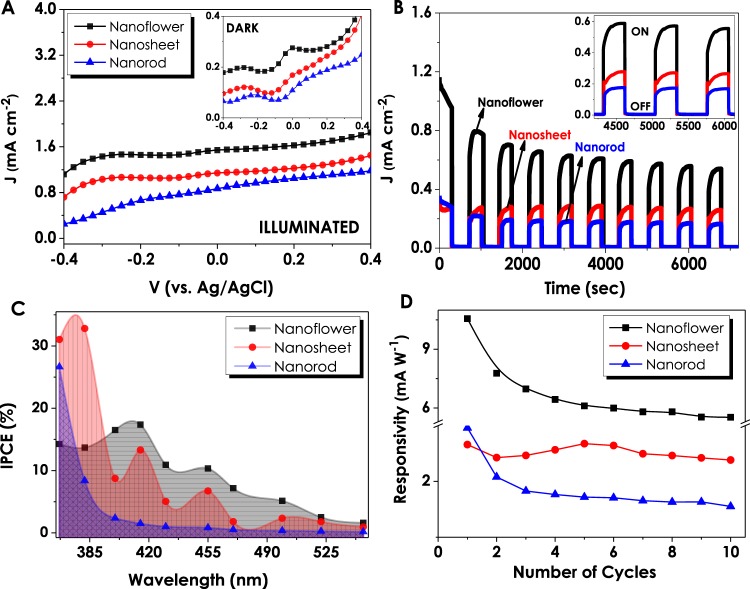
Photoelectrochemical characterizations of three different ZnO nanostructures; (**A**) J-V, (**B**) Stability, (**C**) % IPCE and (**D**) Responsivity values evaluated against the number of stability cycles.

X-Ray Photoelectron Spectroscopy (XPS) has been chosen as a convenient method to investigate and determine the defects of ZnO nanostructures. In accordance with this purpose, O1s spectra of all three samples have been individually assayed. The given spectra of NF and NS structures have been deconvoluted into two fitting Gaussian peaks which correspond to; (1) O1 located at lower binding energy levels (~530.9–531.3 eV) signifying the O^2−^ ions of Zn-O bonding related to the wurtzite structured hexagonal ZnO and (2) O2 residing at higher binding energies (~531.7–533.5 eV) that refer to oxygen-deficient regions of ZnO nanostructures coming from the loosely bound oxygen on the surface (Fig. 1G,H)^40–43^. The relative intensity variations of the O1 and O2 peaks are regarded as a potential tool for the evaluation of oxygen vacancy concentration in ZnO structures. The calculations performed in line with this objective show that the ratio of the higher to lower binding energy peak intensities (O2/O1) are higher for NFs (0.77) and relatively smaller for NSs (0.61) indicating the presence of Vo at higher amounts for NF structures. Large numbers of studies have previously established the relation between oxygen vacancies and optoelectronic as well as photoelectrochemical performances showing that Vo plays the role of the inherent donors in the structure^44–46^. Once breaking away from the ZnO unit cell, the escaped oxygen is balanced with 2e^−^ and oxygen vacancy resulting in the enhanced electrical conductivity due to the increased amount of charge carrier density. Several studies have also proven that the presence of defects such as oxygen vacancies in the crystal lattice trigger the generation of new energy levels between valence and conduction bands having an ultimate crucial effect on light absorption properties^47–50^. In contrary to XPS spectra of NF and NS structures the O1s spectrum of NR ZnO given in Fig. 1I is deconvoluted into three fitting Gaussian peaks denoted as O1, O2 and O3 aligned in the increasing binding energy level orders. These oxygen peaks that have been found to be in compliance with the literature are attributed to the O^2−^ ions in the wurtzite structured ZnO (529.4 eV), Zn-OH bonding (530.9 eV) and chemisorbed oxygen peaks (531.9 eV), respectively^43,51^. The presence of oxygen vacancies has been further confirmed via photoluminescence (PL) and Raman analysis for all three ZnO thin films. Figure 2 shows the PL spectra of NF, NS and NR structures with significant peaks within 370–450 nm and 450–900 nm ranges. ZnO nanostructures grown via CBD process exhibit large numbers of defects of which Zn(OH)_2_ is the main cause of charge entrapment^52^. At the presence of Zn(OH)_2_ sites, holes residing at valence band are withheld in a midgap state as a result of which originate two PL characteristic peaks^53,54^. The first peak occurs due to yellow-green defect emissions at higher wavelengths (≥550 nm) caused by the recombinations between electrons and trapped holes, while the second peak appears due to excitonic emission at UV region (≥380 nm) caused by the Coulombic forces between electron-hole pairs^52^. Although the main cause of green emissions is contradictive yet, the presence of oxygen vacancies has been reported as the main origin of this indication^55–57^. The PL spectra of all three ZnO structures show the presence of the excitonic emission peaks in UV region at approximately the same wavelengths. However, analyzing the differences in green emissions of NF, NS and NR individually, it can be observed that NF structure reveals a single, sharp peak at 550 nm, corresponding to the presence of V_o_ and a shoulder than can also be interpreted as a presence of zinc vacancies (V_Zn_) at comparatively low concentrations. Moreover, the relatively high peak intensities of NF can be assigned to the higher concentration of defects present in the structure. For a deeper investigation of PL analyses and the peaks originating from defects, each spectrum in defect emission region have been deconvoluted into three Gaussian bands; (I) 540–580 nm (O_I_), (II)580–640 nm (O_II_) and (III) 640–740 nm (O_III_), respectively^40^. It has been reported by several studies that the Gaussian peak residing at O_I_ and O_II_ regions correspond to singly charged (V_o+_) and doubly charged (V_o++_) oxygen vacancies, respectively^44,57^. The final Gaussian band at O_III_ region has been found to explain the presence of defects such as V_Zn_ and/or oxygen interstitials (O_i_)^58^. For NF deconvolution band, the intensity of O_I_ originating from V_o+_ is stronger than O_III_ showing the presence of excess oxygen vacancies in the structure. NS structure having a comparatively higher intensity of V_o++_ band also shows a gradual increase in O_III_ intensities. The intensity of O_III_ band dominates in NR in contrast to NF and NS, which corresponds to the presence of V_Zn_ or O_i_ in the structure. For a more apparent comparison, the ratios of intensities of Gaussian bands are summarized in Table S1. It is important to note that these values are in a good agreement with XPS analyses that show a comparatively larger amount of oxygen vacancies in NF structure. Raman analyses have been conducted at the 532 nm laser wavelength, and the resulting spectra for different ZnO nanostructures have been evaluated (Fig. S5). All of the three ZnO samples have generated approximately similar Raman spectra with significant peaks at 330, 440 and 580 cm^−1^. The primary peaks located at 330 and 440 cm^−1^ are assigned to zone boundary phonons E_2H_-E_2L_ and E_2_ mode of ZnO, respectively^59^. Another significant peak at 580 cm^−1^ (E_1_ mode) is contributed by LO mode of ZnO nanostructures which is a nonresonant scattering appearing due to the presence of defects in the structure^59–61^. These defects are mainly reported as oxygen vacancies and/or Zn interstitials in ZnO nanostructures and have a significant contribution to the electron-hole pair generation of these materials. Comparing the intensities of the peaks, NF structure has lower peak intensities, compared to the NR and NS structures, between 300 and 700 cm^−1^ due to the strong fluorescence emission. The PEC performances of ZnO nanostructures have been investigated under 1sun-illumination and dark conditions at the same transmitted amount of light from a solar simulator (Fig. 3A). NF structure resulted in the highest current density (1.54 mA cm^−2^) at 0 V_bias_ (versus Ag/AgCl) that has been attributed to a better crystallinity and oxygen vacancies (Vo) contained in its structure. In accordance with the EDAX analyses, NS and NR structures generate lower current densities (1.14 and 0.8 mA cm^−2^, respectively) owing to an excess amount of oxygen contained in them. Stability tests have been performed under consecutive illuminated and dark cycles at 0 V_bias_ (versus Ag/AgCl) in order to provide an insight into the performance stabilities of thin films (Fig. 3B). Similar to the J-V performances obtained from voltammetry analyses, NF structure generated the highest current density in comparison to the rest of the structures and showed decent stability throughout the 10 cycles. Although the current generation performances of NS and NR are lower than that of NF, the stability they demonstrated during 7200 seconds of consecutive on/off cycles has been observed to be supremely long-lasting.

In addition, incident photon-to-current conversion efficiencies (IPCE) for all three films have been evaluated under the monochromatic light illumination condition. The wavelength dependent IPCE calculations have been done according to the equation given below:

1 $$IPCE\,( \% )=\frac{{I}_{ph}\,(A)}{P(W)}\times \frac{1240}{\lambda (nm)}\times 100$$

Here, *I*_*ph*_ represents photocurrent density, λ stands for the wavenumber of the monochromatic light and *P* is the power density of the light source which varies according to the monochromatic light. The results have been evaluated for three ZnO morphologies within the UV-visible region. The obtained results show a strong UV responsivity of ZnO nanostructures resulting in high current densities at illuminated conditions and almost a negligible response at dark conditions (Fig. 3C). Although NS and NR structures have been observed to be more responsive at 367 nm wavelengths, it has been decided that interpreting the whole performance of 10 cycles would be a much more sound judgment. Therefore, throughout the 10 different sets of IPCE cycles, NF demonstrated the highest and most stable performance. Responsivity data, which is calculated according to the given formula, *R* = (*J*_*ill*_ − *J*_*dark*_)/*P*_*light*_ demonstrates electrical responses of ZnO photoelectrodes to the light. Figure 3D gives the highest responsivity value of NF which as a consequence of stability test has decreased gradually up to the 4th cycle and then remained almost constant. Similarly, the responsivity of the NR was almost constant after the 4^th^ cycle. Conversely, the responsivity of NS electrodes, although being almost stable through the whole 10 cycles, were much lower than that of NFs.

### Properties of strained ZnO electrodes

**Figure 4:**
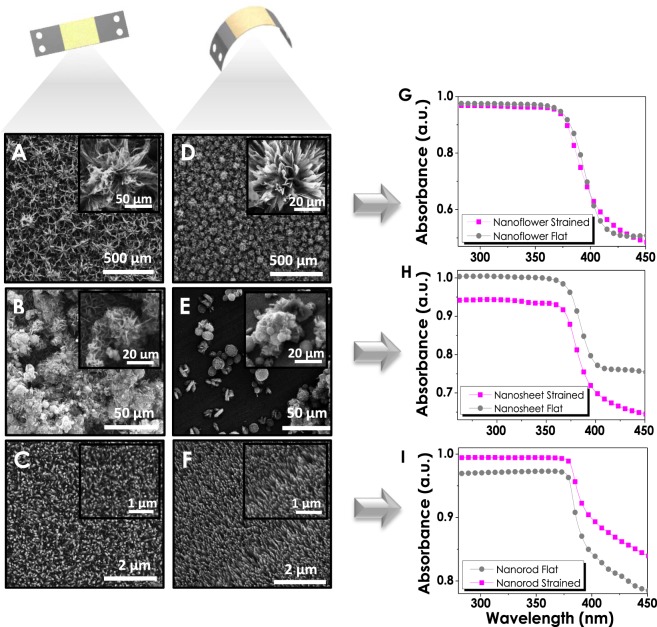
Comparison between flat and strained SEM images of (**A**–**D**) Nanoflower, (**B**–**E**) Nanosheet and (**C**–**F**). Nanorod; Consequent optical properties (absorbance) of (**G**–**I**). NF, NS, and NR consequently.

The incorporation of ZnO photoelectrodes to different curvature systems has been the primary motivation for the SS foil utilization in these experiments. Therefore, flexibility, the degree of straining and strength of ZnO electrodes are important parameters for portable systems and curved designs. Figure 4 gives a fair comparison between the SEM images of ZnO nanostructures deposited on stainless steel electrodes prior to and after the straining of the overall structure. It is important to mention that the foils have been strained after thin film deposition steps forcing the structural changes to occur in ZnO nanostructures. Changes in NF structure as a result of straining are clearly shown in Fig. 4A,D, where the distorted flower-like structures have turned in to more exposed shapes. The significant changes in petals of NFs after the effect of straining reveals the rectangular shapes that could not be observed without being bent. On the other hand, NS structures (Fig. 4B,E) showing a continuous film formation throughout the electrode prior to straining have turned into a balder surface with a few NS-like structures sprinkled around. This indicates their poor degree of adhesion in comparison to those of NFs resulting in delamination after being strained. Additionally, NRs given in Fig. 4C,F, demonstrate a distinct inclining in their structures making them more oriented and unidirectional with pointed tips. The transformation of such disorganized NR arrays into the neat and ordered structure has been expected to have a severe impact on the optical properties of the material.

Optical characterizations of ZnO electrodes have been performed via UV/Vis spectra, and absorbance values have been evaluated. NF structures due to being the densest ZnO films among all three have not shown a severe change after straining (Fig. 4G). A noticeable decrease can be observed for NS structures which are in accordance with SEM images approving  the delamination from the surface (Fig. 4H). However, for NR structures a distinguishable increase in reflectance can be correlated with the increase in light absorption due to the change in the structural orientation and transformation into a more organized structure (Fig. 4I).

**Figure 5:**
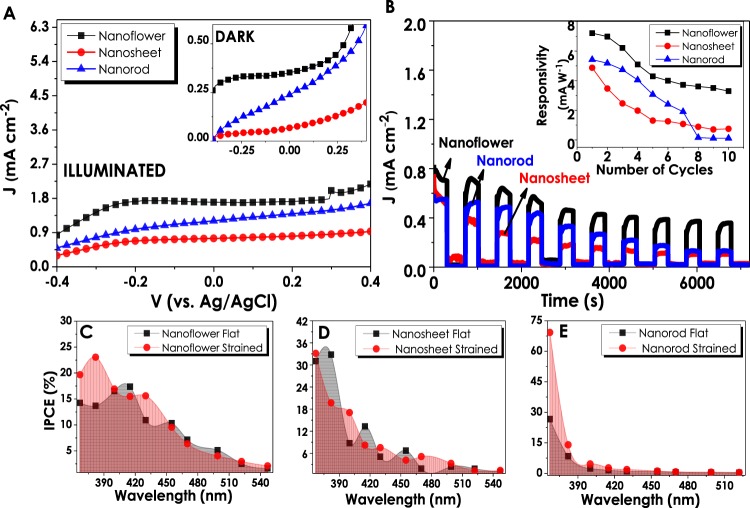
Photoelectrochemical performances of ZnO nanostructures after the straining: (**A**) J-V data for the illuminated and dark ZnO thin films; (**B**) stability and responsivity values of strained ZnO films over 7200 seconds; Individual comparison of % IPCE results versus Ag/AgCl electrode for (**C**) Nanoflower (**D**) Nanosheet and (**E**) Nanorod electrodes, respectively.

The photoelectrochemical performances of ZnO nanostructures after straining effect have been investigated inside a PEC cell with Pt and Ag/AgCl being counter and reference electrodes, respectively. Figure 5A illustrates the J-V responses of strained ZnO nanostructures vs. Ag/AgCl reference electrode. In compliance with the SEM and absorbance values of NS structures, the current density values obtained from these electrodes have shown a severe decrease from 1.14 to 0.74 mA.cm^−2^ at 0 V (vs. Ag/AgCl) after straining. Conversely, NF and NR structures have demonstrated an improvement in the amount of generated current density. For NF structures, the J values have increased from 1.54 to 1.70 mA cm^−2^ at 0 V_bias_ (vs. Ag/AgCl) after straining. The most distinguishable performance increase (from 0.87 to 1.2 mA.cm^−2^) has been observed for strained NR structures, which is about 1.4 times higher than the J value of flat one. The stability and responsivity results evaluated for all three structures demonstrate a significant increase in NR performance which is in agreement with SEM and %Absorbance data. The change in the structure orientation of NRs has turned to have a serious effect on the electrical responsivity values of electrodes to light, generating higher currents at the first six cycles under the same power input. Nevertheless, NF electrodes still exhibited the highest current densities through 10 cycles of stability and responsivity tests even after straining, showing the best performance in all kinds of photoelectrochemical analysis (Fig. 5B). As can be seen in the inset figure of Fig. 5B, unlike its flat forms, strained NRs showed a better responsivity than NSs up to eighth cycle. Also, the responsivity of the NRs increased significantly with straining compared to their flat form.

Figures 5C–E compare the IPCEs of ZnO nanostructures in their flat and strained forms individually. The IPCE% values evaluated for all three films after being strained demonstrate the improving effect of mechanical deformation on the film performances. Especially, NR structures having the lowest IPCE% values in their flat forms have increased by 106% after straining (Fig. 5E). However, it is important to underline the fact that the mentioned improvement in efficiency took place in UV region signifying the enhancement of UV responsivity of NR structures. Besides, NRs displayed better performance compared to NSs, also related to the increase in surface area that contributes to the generation of electron-hole pairs. After straining, IPCE of NFs have increased by 10.2% and reached up to approximately 23%. NR electrodes had the most remarkable improvement in efficiency from 27 to 69% while the IPCE of NSs has decreased by approximately 5.5%. The summary of the IPCE values obtained from both flat and strained ZnO electrodes and the comparison with the highest efficiencies reported in the literature are given in Table 2. As can be seen in this table, our NR electrodes after straining had one of the highest performance reported in the literature so far.

**Table 2:** Comparison of IPCE values of fabricated nanostructured thin film photoanodes.

ZnO Nanostructures	Electrolyte	J_max_ (mA cm^−2^)	IPCE (%)	Ref.
Nanorod	0.25 M Na_2_S/0.35 M Na_2_SO_3_	0.50	26% at 300 nm	^68^
Nanowire	0.25 M Na_2_S/0.35 M Na_2_SO_3_	0.50	8% at 325 nm	^69^
Nanowire	0.2 M Na_2_SO_4_	1.20	52% at 370 nm	^70^
Nanocluster	0.5 M Na_2_SO_4_	0.56	10% at 300 nm	^71^
Nanotree	0.5 M Na_2_SO_4_	0.67	12% at 300 nm	^71^
TiO_2_ – ZnO Nanorod	PBS	0.83	32% at 350 nm	^72^
ZnO Nanorod	0.5 M Na_2_SO_4_	0.15	30% at 380 nm	^73^
Flat Nanoflower	0.25 M Na_2_S/0.35 M Na_2_SO_3_	1.54	18% at 420 nm	This work
Flat Nanosheet	0.25 M Na_2_S/0.35 M Na_2_SO_3_	1.14	33% at 367 nm	This work
Flat Nanorod	0.25 M Na_2_S/0.35 M Na_2_SO_3_	0.87	27% at 367 nm	This work
Strained Nanoflower	0.25 M Na_2_S/0.35 M Na_2_SO_3_	1.70	24% at 390 nm	This work
Strained Nanosheet	0.25 M Na_2_S/0.35 M Na_2_SO_3_	0.74	34% at 390 nm	This work
Strained Nanorod	0.25 M Na_2_S/0.35 M Na_2_SO_3_	1.20	69% at 390 nm	This work

### EIS measurements of ZnO nanostructures

**Figure 6:**
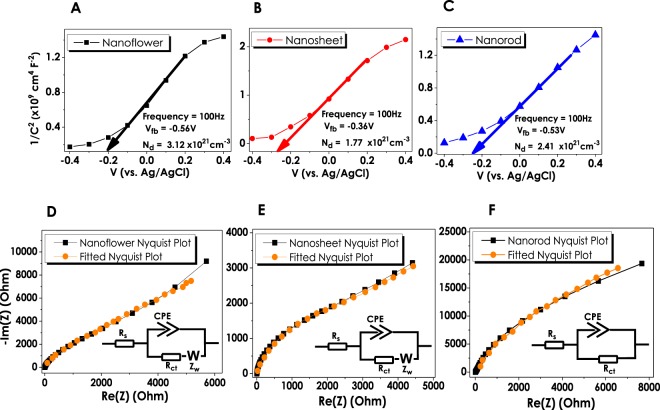
Photoelectrochemical EIS Measurements: (**A**–**C**) Mott-Schottky plots for NF, NS and NR structures with corresponding V_fb_ and N_d_ data, respectively; (**D**–**F**) Nyquist plots of NF, NS and NR with the obtained fits from relevant equivalent circuit models.

Electrochemical Impedance Spectroscopy (EIS) measurements of three different ZnO nanostructured thin films have been conducted in order to understand the intrinsic material properties such as flat band potential (V_fb_) and majority carrier concentration (N_d_). The Mott-Schottky (MS) analyses have been performed under dark conditions at 0.25/0.35 M Na_2_S/Na_2_SO_3_ environment and 100 Hz. Figure 6A–C represent the MS data of NF, NS and NR structures, respectively. The x-intercept obtained from the extrapolation of the linear region of 1/C^2^ to zero gives the flat band potential of the corresponding ZnO nanostructure^62,63^. The V_fb_ values of NF, NS, and NR structures have been obtained as −0.56 V, −0.36 V and −0.53 V (vs. Ag/AgCl), respectively. The positive slopes (n-type ZnO) also derived from the linear regions of all three plots correspond to the carrier concentration which has been calculated as 3.12 × 10^21^, 1.77 × 10^21^ and 2.41 × 10^21^ cm^−3^ for NF, NS and NR, respectively (Ɛ = 10, Ɛ_0_ = 8.85 × 10^−14^ F cm^−1^)^62^. Although the carrier concentration evaluated for all ZnO nanostructures were very close to each other, ZnO NF sample had a slightly higher N_d_ value, which was in a good agreement with the obtained PEC measurements and IPCE values.

Nyquist analysis, a frequency dependent handy EIS characterization method for the determination of circuit elements, has been performed in order to determine the electrochemical impedance data for ZnO nanostructures at different excitation frequencies (Fig. 6D–F). The obtained plot is generally fitted into an equivalent circuit (inset Fig. 6D–F) that contains passive circuit components (resistor, capacitor, etc.)^63^. EIS plots for NF and NS thin films have been fitted to Randles equivalent circuit representing the diffusion dominant behavior where Warburg impedance is observed^64^. The circuit consists of a series resistance (R_s_) symbolizing the resistance of the electrolytic solution, constant phase element (CPE) connected in parallel with Faradaic charge transfer resistance (R_ct_) and the Warburg element. The inclusion of the Warburg element into the circuit is due to the linear, tilted response with approximately 45° phase shift observed for NF and NS electrodes^64^. The obtained values for each of the circuit elements have been specified in Table 3. As can be seen in this table, the equivalent circuit of NR thin films had no Warburg element indicating a kinetic-controlled charge transfer mechanism. The solution resistance, R_s_, of all three samples is very low and comparable to each other. On the other hand, a drastic change in the charge transfer resistance, R_ct_, depending on the morphology of the ZnO has been observed. R_ct_ of the NS and NR electrodes was 3.2 and 61 kΩ, respectively. On the other hand, NF thin films had very low R_ct_ of 40.7 Ω. The higher photocurrent density of the NF thin films, obtained from PEC measurements, can be attributed to this very low R_ct_ value. Previously, charge transfer resistance of  3 Ω has been reported for the ZnO thin films deposited on the graphene^65^. Similarly, Z. Han *et al*. have reported the R_ct_ of 211.6 Ω for the ZnO with nano flower-rod morphology^66^. It has been suggested that the oxygen vacancies restrain the electron-hole recombination and due to the low R_ct_, charges could move quickly through ZnO^66^.

**Table 3:** Corresponding values of circuit elements for NF, NS and NR structured ZnO thin films.

ZnO Morphologies	Circuit Elements				
Nanoflower	**R**_**s**_ (**Ω**)	**R**_**ct**_ (**Ω**)	**CPE** (**µF.s**^(**α−1**)^)	**a**	**W** (**Ω.s**^**−0.5**^)
	7.2	40.7	45.2	0.9	6500
Nanosheet	**R**_**s**_ (**Ω**)	**R**_**ct**_ (**Ω**)	**CPE** (**µF.s**^(**α−1**)^)	**a**	**W** (**Ω.s**^**−0.5**^)
	5.7	3200	79.5	0.8	1900
Nanorod	**R**_**s**_ (**Ω**)	**R**_**ct**_ (**Ω**)	**CPE** (**µF.s**^(**α−1**)^)	**a**	
	4.1	61 × 10^3^	62.3	0.9	

### FDTD simulation of straining effect

**Figure 7:**
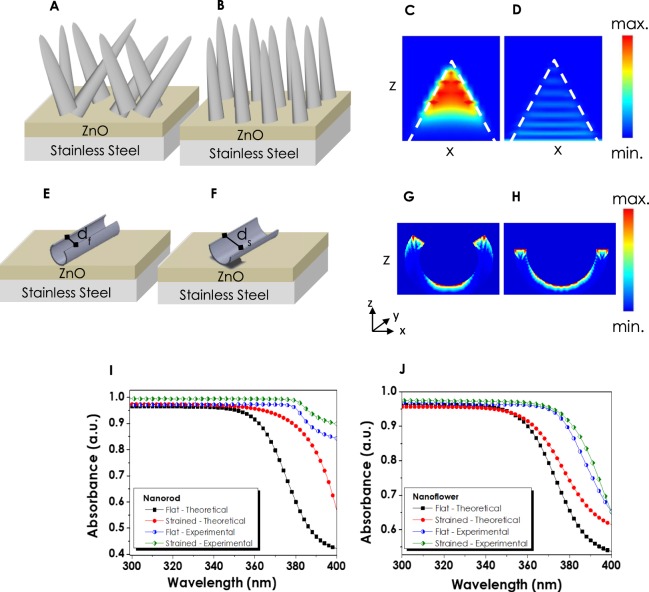
ZnO nanorods in (**A**) flat and (**B**) strained forms grown on ZnO seed layer; Absorption maps of nanorods at (**C**) 300 nm and (**D**) 450 nm wavelengths; Representation of a single nanoflower petal in (**E**) flat and (**F**) Strained forms with corresponding (**G**–**H)** Absorption maps; Comparison of absorbance values obtained from experimental and numerically calculated ZnO (**I**) nanorods and (**J**) nanoflowers.

The effects of straining on the light absorption of NF and NRs have been numerically investigated via Lumerical FDTD software in order to expound the reason behind the enhanced performances. The determined structural parameters for ZnO NR have been specified as 1000–1500 nm length, 50 nm radii and 80–90 μm^−1^ NR area density on average. The SEM images revealed that the shapes of NRs are mainly conical or pruned conical rather than cylindrical. Therefore, simulations have been performed for NRs with conical tips (Fig. 7A,B). For the numerical investigations, the complex refractive index of ZnO has been extracted from an earlier experimental study^67^, and the change in complex refractive index with wavelength has been demonstrated in Fig. S6 in Supplementary Information. In addition, changes in the absorption of thin films as a result of straining have been compared with the experimental outcomes for both NR and NF thin films.

The structure used in the numerical calculations given in Fig. 7A consists of a planar stainless steel substrate, 150 nm thick planar ZnO seeding layer and long NR-like formations at flat conditions. Moreover, the SEM images (Fig. 4) revealed that the NR thin films in their flat form had been grown on the seeding layer randomly (Fig. 7A). On the other hand, each NR orients perpendicular to the surface and parallel to the neighboring NRs as a result of straining (Fig. 7B). For the numerical calculations for flat conditions of NRs with various lengths (1000 nm to 1500 nm) and the upper radii (0 nm to 50 nm), the angle between wire and surface has been altered from 30° to −30° in the z-axis. As a result of straining, each NR has been demonstrated to be positioned in parallel to each other. The remarkable increase in the performances of NRs has been attributed to this obvious change in their arrangements which can be seen from Fig. 7I. The absorption maps of NRs at 300 nm and 450 nm have been extracted in order to determine the regions of NRs where the light is absorbed. From the Fig. 7C,D, it can be interpreted that the light is absorbed mainly in the upper parts of the NRs at small wavelengths, while for higher wavelength values the intensity of absorption decreases and occurs throughout the whole NR body. Furthermore, the effect of the angle of incidence also has been given in Fig. S7 in Supplementary Information. It has been observed that the angle of incidence had no significant effect on the absorption.

The absorption simulations and numerical analyses for NF formations have raised some difficulties due to a more complex and 3D structure. For the proper investigations of changes in the light absorption of NFs, a simulation based on a single petal of a flower has been conducted. From the obtained SEM images (Fig. S8 in Supplementary Information) it has been observed that at flat conditions, the petals of NFs resemble an entwisted structure (d_f_ = 1.6 µm) from their short edges which are further unwrapped (d_s_ = 4 µm) as a result of straining (Fig. 7E–F). This structural diversity has been adapted into the FDTD simulations where the obtained absorption maps demonstrate the regions at which light absorption occurs. In a wrapped petal structure, the inner surface area is impenetrable for light causing a loss in performance. After being strained, the blossomed petal-like formations disclose their inner surface area contributing more to the light absorption which is clearly seen from the absorption maps in Fig. 7G,H. The changes in the amount of absorbed light numerically calculated via FDTD are also given in Fig. 7J which are in accordance with the experimentally obtained absorbance values. The absence of identity between the whole NF structure and the simulated single petal and the differences between the theoretical and experimental refractive indices has been regarded as the main constraints for NF simulations. However, the proximity of the absorbance values for both theoretical and experimental data approves the fact that the act of straining has an ultimate impact on the morphology and consequently the absorption of ZnO thin films. As evidenced by both FDTD and the UV-VIS analyses, the optical absorption of the ZnO –NR, and NF increased with the straining. With this increase, it is possible to generate more light-induced carriers, which has a direct effect on the enhancement of the photoelectrochemical performance.

### Cyclic deformation of ZnO electrodes

**Figure 8:**
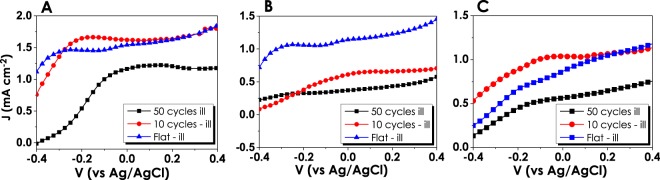
J-V data for 10 and 50 straining cycles applied on (**A**) Nanoflower, (**B**) Nanosheet and (**C**) Nanorod, respectively.

**Figure 9:**
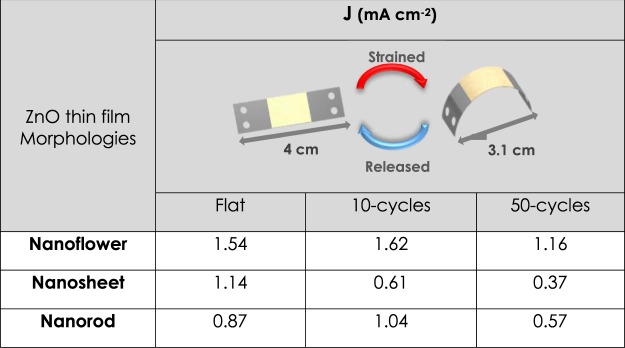
Summary of photocurrent densities of ZnO electrodes at 0 V_bias_ (vs. Ag/AgCl) after consecutive straining cycles.

Cyclic deformation analyses aimed to give an insight into the durability of electrodes to continuous deformation cycles. For this purpose, consecutive straining cycles have been applied where ZnO electrodes have been strained and flattened under the applied force. Straining cycles on flexible electrodes have been repeated 10 and 50 times and the changes in photocurrent densities have been recorded (Fig. 8). The effect of 10 cycles of stain varied for each film relatively. As for NF and NR structures, ten cycles of straining did not cause a critical fall in the performance and even resulted in an increase in the current density (Fig. 9). In other words, NR and NF ZnO thin film electrodes showed better performance compared to their flat form, up to 10 cycles of deformation. However, with the increase in the number of straining cycles the delamination of all films from the surface has increased, resulting in performance decay. For the NF and NR electrodes after 50-cycle of straining, we calculated a 25 and 34% decrease in the current densities, respectively. Moreover, NS electrodes, which already had the lowest amount of generated photocurrent, have shown terrific performance decay after cyclic straining. In order to better understand the durability of the thin film electrodes, further straining have been applied up to 2000 cycles. As given in Table S2, complete delamination from the surface was observed for the NS electrodes for 100 cycles of straining. On the other hand, after 100 cycles NF and NR electrodes had the current density of 0.29 and 0.57 mA.cm^−2^, respectively. At 500-cycles both NS and NF electrodes showed no response to the illumination. Conversely, NR electrodes were still responsive to the light even after 2000 straining cycles. The current density of 0.41 mA.cm^−2^ has been measured for the NR electrode, deformed under 2000-cycle. In other words, NR electrodes tolerated the 2000 cycles of straining with 47% performance loss in photocurrent density. Therefore, it has been concluded that the ZnO-NF and -NR electrodes are very suitable for the utilization of them in a PEC reactor having curvatures.

## Conclusions

Three different ZnO nanostructures have been successfully synthesized on stainless steel foils by a chemical bath deposition technique. Morphological, structural, optical and photoelectrochemical performances of flexible electrodes have been tested under flat and strained conditions. Performance of NF-like structures is superior to other morphologies in their flat forms. A very high responsivity of 6 mA W^−1^ for NF thin films has been endured after ten on-off cycles during the photoelectrochemical performance test, indicating the better stability of them compared to the NR and NS thin films. The superior photoelectrochemical performance of the NF thin films could be attributed to the better crystallinity indicated by the XRD analysis and oxygen vacancies implied by Raman, PL, EDX, and XPS results. Additionally, electrochemical impedance spectroscopy studies indicated that the charge transfer resistance of the NF thin films was lower than that of NR and NS thin films. This could be attributed to the enhanced electron-hole separation in the NF thin films due to the presence of the oxygen vacancies.

Analyzing the properties of strained electrodes, a distinguishable change in morphology has been observed for all nanostructures which resulted in a performance increase of NF and NR electrodes. Conversely, due to the delamination problem of NS structures, performance decay has been detected. The maximum IPCE of 69% has been calculated for the NR thin film electrodes after straining. According to our best knowledge, this efficiency is one of the best-reported performances for bare ZnO electrodes. In order to enlighten this remarkable enhancement in the performance, we examined the morphological and optical properties of the strained ZnO electrodes. Besides FDTD simulations have been carried out to examine the optical properties of flat and bent ZnO electrodes. As confirmed by FDTD simulations, the formation of more hierarchical ZnO NR arrays resulted in the increase in the light absorption. Finally, consecutive cyclic deformations measuring the durability of ZnO electrodes showed that NR structures are highly tolerant to mechanical stress which confirms their adaptability to cylindrical, tubular and mobile systems.

## Methods

### Fabrication of ZnO nanostructures

Prior to use as a substrate for ZnO seeding layers, the SS foils have been cleaned in an ultrasonic bath containing acetone, alconox solution, deionized water and 5% v/v acetic acid solution, respectively. Following to cleaning process, the substrates were completely dried with nitrogen gas. The zinc oxide (ZnO) seeding layers have been deposited on the SS substrates by RF magnetron sputtering (Vaksis Midas PVD-MT/2M2T, Ankara, Turkey). The deposition was maintained at a chamber pressure of 7.6 × 10^−6^ Torr in 99.999% purity of the argon gas environment. The plasma discharge has been generated constantly at 60 W for 9 minutes. The coated substrates have further been annealed in Rapid Thermal Annealing (RTA) furnace (Vaksis, RTAP Handy Tube) at 300 °C for 30 minutes.

ZnO nanostructures in different morphologies such as NF, NS, and NR have been grown on the ZnO seeding layer coated substrates via CBD method by altering the anionic species. For the synthesis of NF and NS structures, an aqueous solution containing 1.0 M of urea and 0.05 M of Zn(C_4_H_6_O_4_.2H_2_O) and (Zn(NO_3_)_2_.6H_2_O) have been prepared, respectively. The pH values of both solutions have been adjusted to 4.5 strictly by using acetic acid for NF and nitric acid for NS morphologies. The deposition quality of NF structure strongly depends on the pH of the solution showing that the process is highly sensitive to the number of hydronium ions. The substrates have been immersed in chemical bath solutions vertically and kept in an oven at 80 °C for 3 hours. Further, samples have been withdrawn from the solution, washed with deionized water and calcinated at 300 °C for 30 minutes. For NR synthesis, (Zn(NO_3_)_2_.6H_2_O) (0.1 M) has been dissolved in deionized water and ammonium hydroxide of (2% v/v) has been added to the solution. The CBD process for ZnO NR formation is maintained at 80 °C, 1 hour. The coated samples have been further annealed at 300 °C for 30 minutes.

### Straining Analyses and Cyclic Deformation Tests

During the straining tests, SS foils have been subjected to mechanical deformations where they have been strained by adjusting the distance between two ends of SS foil to a certain fixed value. The initial dimensions of SS foil prior to straining were 10 × 40 mm (width × length) which has decreased to 10 × 31 mm after straining. Cyclic deformation tests have been conducted with 10 and 50 times straining and relaxation periods (Fig. 9).

### Material Characterization

Morphological analyses have been performed via scanning electron microscopy (QUANTA 400 F Field Emission SEM). Images of both flat and strained ZnO films have been captured in order to determine the effect of deformation. The crystal structures of synthesized ZnO nanostructures have been determined by X-Ray Diffraction (XRD) analysis. XRD analysis has been performed via the PANalytical/Philips X’Pert MRD system. The flexibility of ZnO films have also been tested, and the changes in film characteristics have been investigated deeply. UV/Vis (Perkin Elmer, Lambda 650 S) has been used for the reflectance measurements in order to determine the optical changes after straining.

### Electrochemical characterization

The photoelectrochemical (PEC) performances of ZnO films have been tested by using a standard three-electrode cell where ZnO films, Platinum (Pt) and Ag/AgCl have been used as working, counter and reference electrodes, respectively. The PEC performances have been tested in aqueous solution (pH value 12) containing 0.25 M of Na_2_S and 0.35 M of Na_2_SO_3_. The current density of samples versus the applied potential has been analyzed by using Gamry750 Potentiostat/Galvanostat/ZRA. The active area of analyzed samples was 1 cm^2^. The measurements have been performed at room temperature, and J-V curves of samples have been evaluated for both illuminated and dark conditions. As the light source for IPCE measurements, a monochromatic light source has been used (FemtoTera, Femto-RD5). Current density versus voltage measurements have been carried out by Lot Oriel Solar simulator equipped with 150 W Xenon Lamp.

## Supplementary information

Electronic Supplementary Information
