# Supplementary material for: Investigation of Strain Effects on Photoelectrochemical Performance of Flexible ZnO Electrodes
Source: Sci Rep. 2019 Jul 29;9:11006. doi: 10.1038/s41598-019-47546-1 (PMC6662888; doi:10.1038/s41598-019-47546-1)
Supplement: Supplementary file 1 — Electronic Supplementary Information [file 41598_2019_47546_MOESM1_ESM.pdf]

Electronic Supplementary Information

Investigation of Strain Effects on Photoelectrochemical Performance of Flexible ZnO Electrodes

Nazrin Abdullayeva[1], Cigdem Tuc Altaf[1], Merve Mintas[2], Ahmet Ozer[3], Mehmet Sankir[1,2,*], Hamza Kurt[1, 2, 3], Nurdan Demirci Sankir[1,2,*]

[1]Micro and Nanotechnology Graduate Program, TOBB University of Economics and Technology, Sogutozu Caddesi No 43 Sogutozu 06560 Ankara, Turkey

[2]Department of Materials Science and Nanotechnology Engineering, TOBB University of Economics and Technology, Sogutozu Caddesi No 43 Sogutozu 06560 Ankara, Turkey

[3]Department of Electrical and Electronics Engineering, TOBB University of Economics and Technology, Sogutozu Caddesi No 43 Sogutozu 06560 Ankara, Turkey

*Corresponding authors: nsankir@etu.edu.tr; msankir@etu.edu.tr

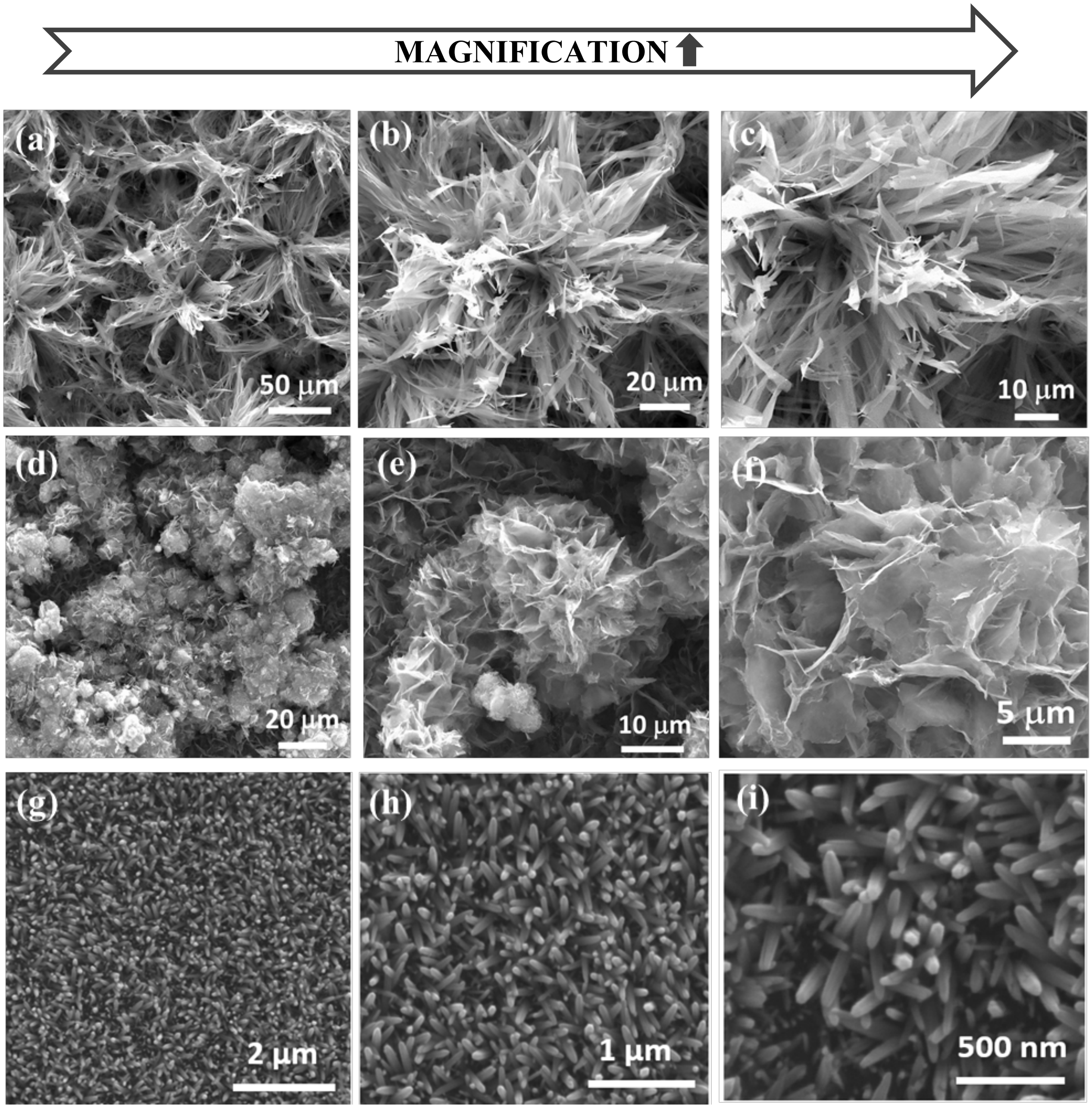

Fig. S1. SEM images of **(a) – (c)** Nanoflower, **(d) – (f)** Nanosheet and **(g) – (i)** Nanorods at different magnifications

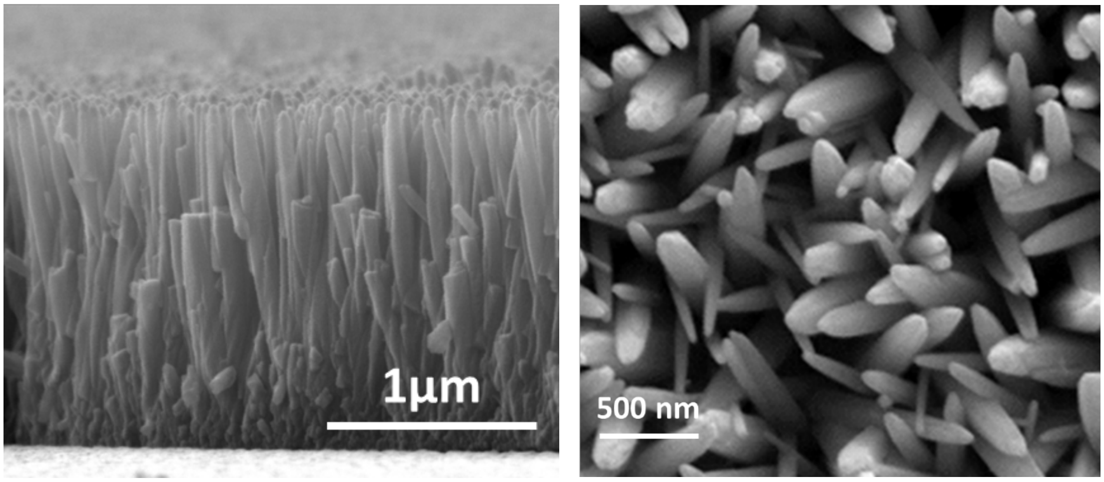

Fig. S2. Cross sectional and surface SEM images of ZnO nanorods.

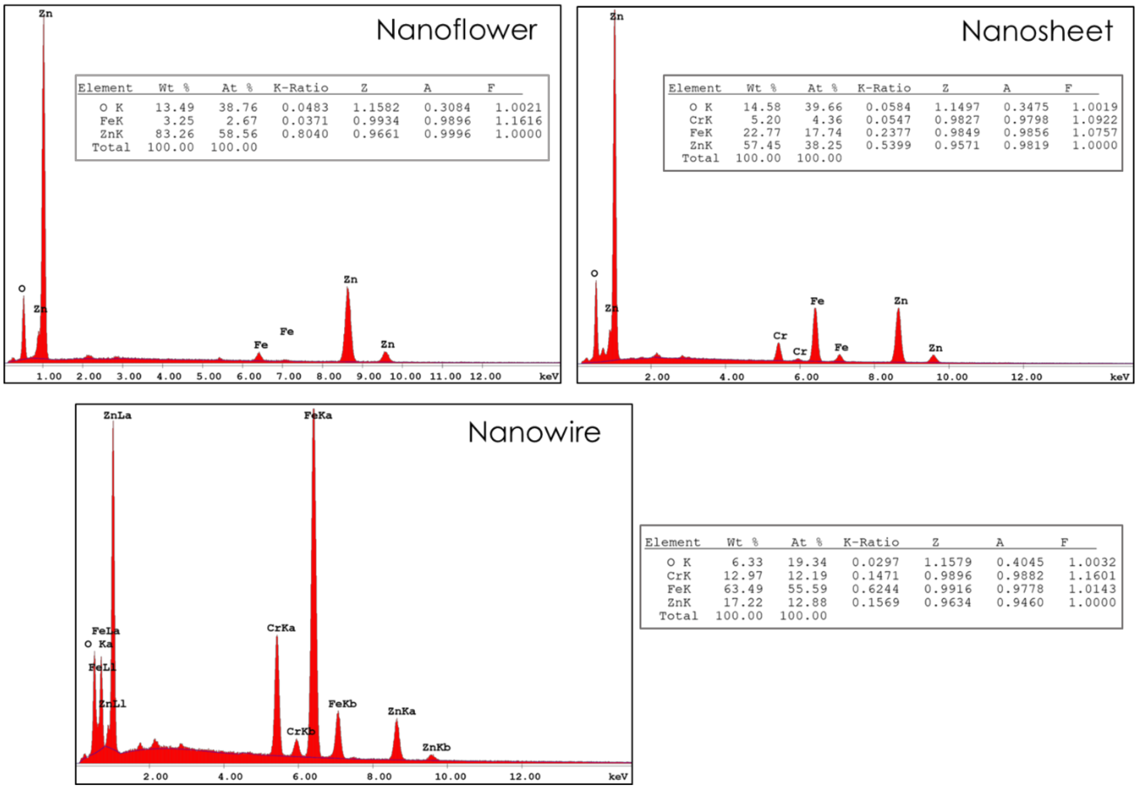

Nanoflower

Element	Wt %	At %	K-Ratio	Z	A	F
O K	13.49	38.76	0.0483	1.1582	0.3084	1.0021
FeK	3.25	2.67	0.0371	0.9934	0.9896	1.1616
ZnK	83.26	58.56	0.8040	0.9661	0.9996	1.0000
Total	100.00	100.00				

Nanosheet

Element	Wt %	At %	K-Ratio	Z	A	F
O K	14.58	39.66	0.0584	1.1497	0.3475	1.0019
CrK	5.20	4.36	0.0547	0.9827	0.9798	1.0922
FeK	22.77	17.74	0.2377	0.9849	0.9856	1.0757
ZnK	57.45	38.25	0.5399	0.9571	0.9819	1.0000
Total	100.00	100.00				

Nanowire

Element	Wt %	At %	K-Ratio	Z	A	F
O K	6.33	19.34	0.0297	1.1579	0.4045	1.0032
CrK	12.97	12.19	0.1471	0.9896	0.9882	1.1601
FeK	63.49	55.59	0.6244	0.9916	0.9778	1.0143
ZnK	17.22	12.88	0.1569	0.9634	0.9460	1.0000
Total	100.00	100.00				

Fig. S3. EDAX results of ZnO thin films

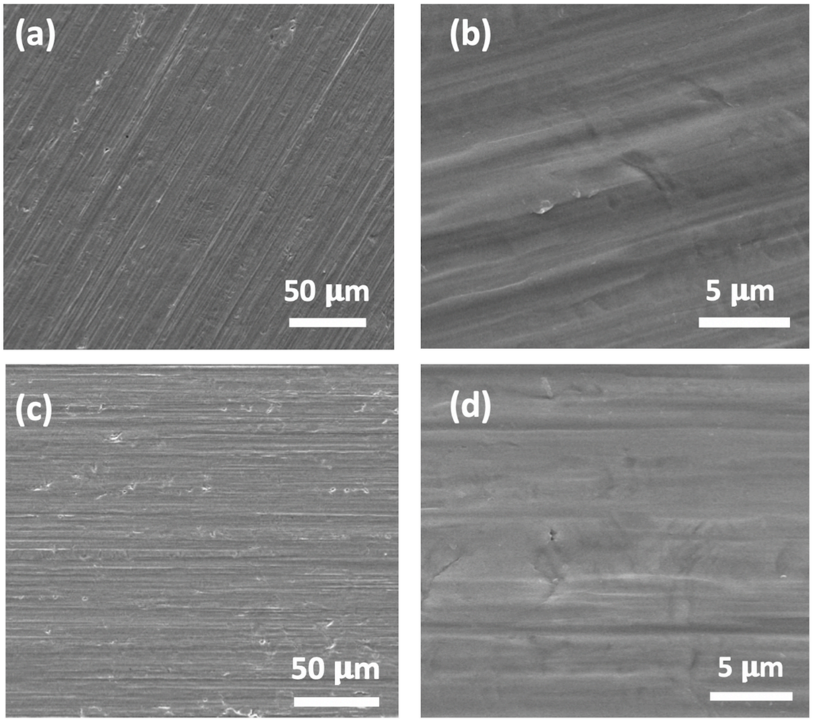

Fig. S4. SEM images and EDAX results of **(a)-(b)** bare SS foil, and **(c)-(d)** ZnO seeding layer deposited SS foil.

Table S1. Peak intensity ratios obtained from PL deconvolutions of ZnO nanostructures

ZnO Morphologies	O_I/O_{III}	O_{II}/O_{III}
Nanoflower	1.22	0.50
Nanosheet	0.73	1.10
Nanorod	0.94	0.52

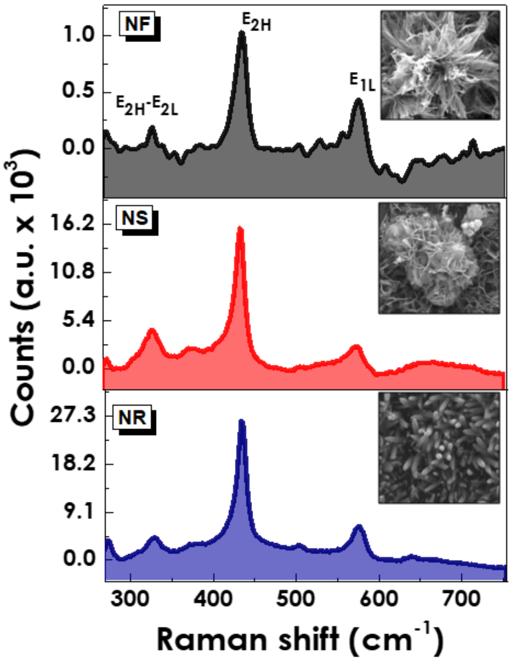

ZnO Morphologies	E_{1L}/ E_{2H} intensity ratios
Nanoflower	0.41
Nanosheet	0.15
Nanorod	0.23

Fig. S5. Raman spectra for NF, NS and NR ZnO nanostructures with corresponding characteristic peaks.

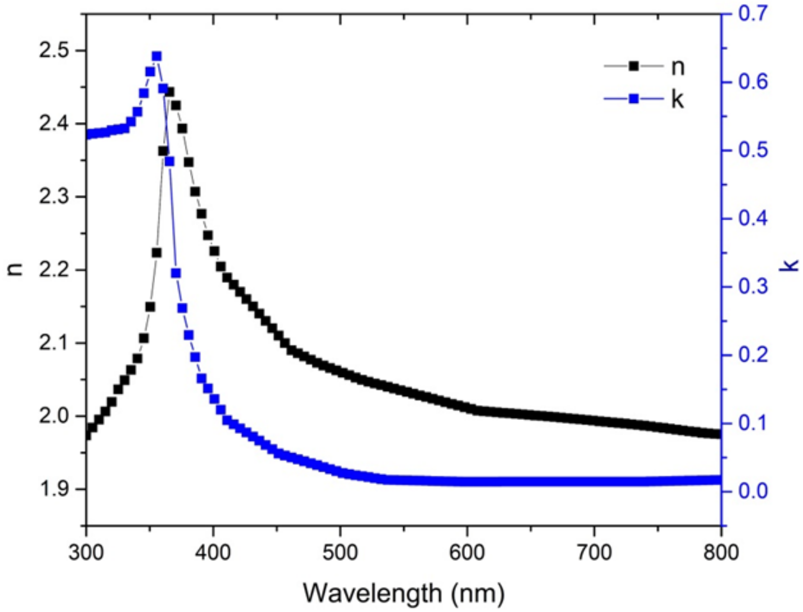

Fig. S6. Complex refractive index of ZnO

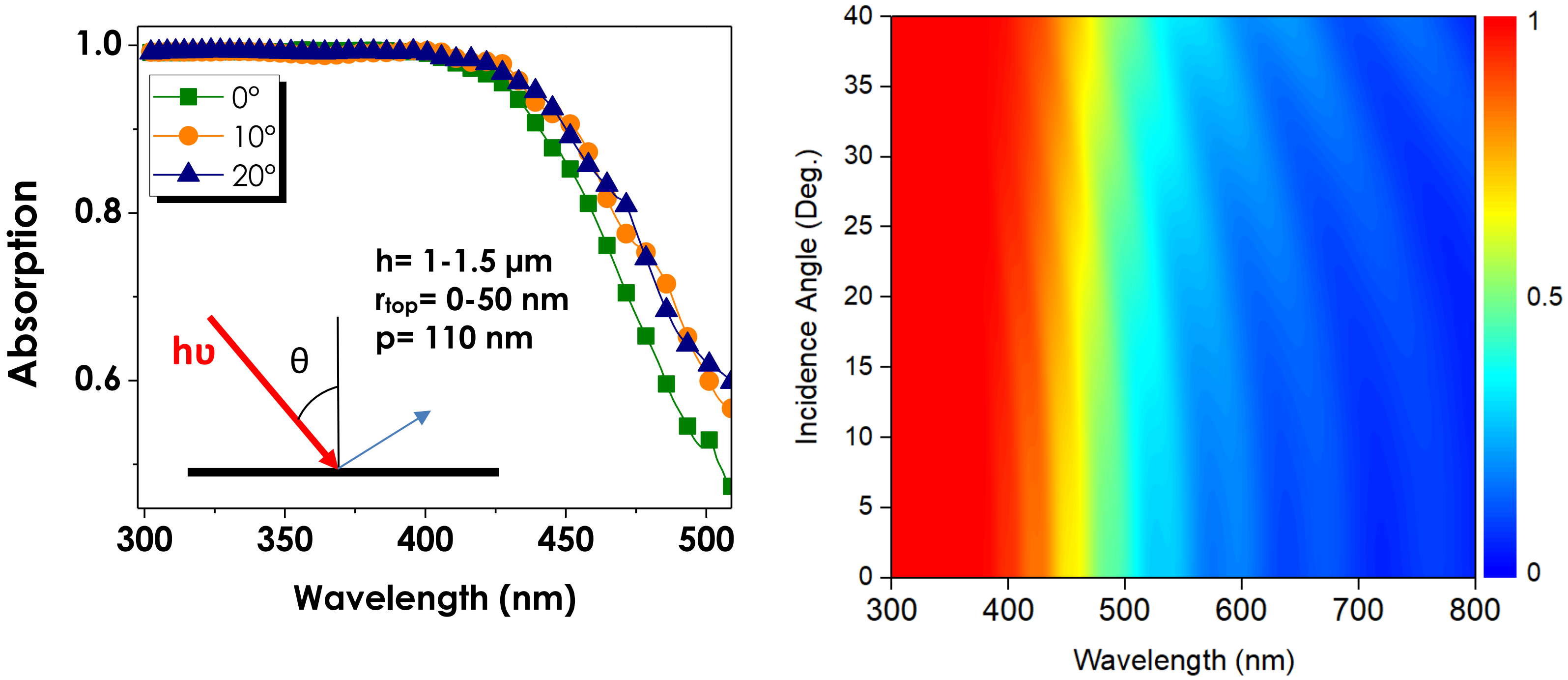

Fig. S7. The dependence of the absorption of ZnO NWs on the angle of incident light

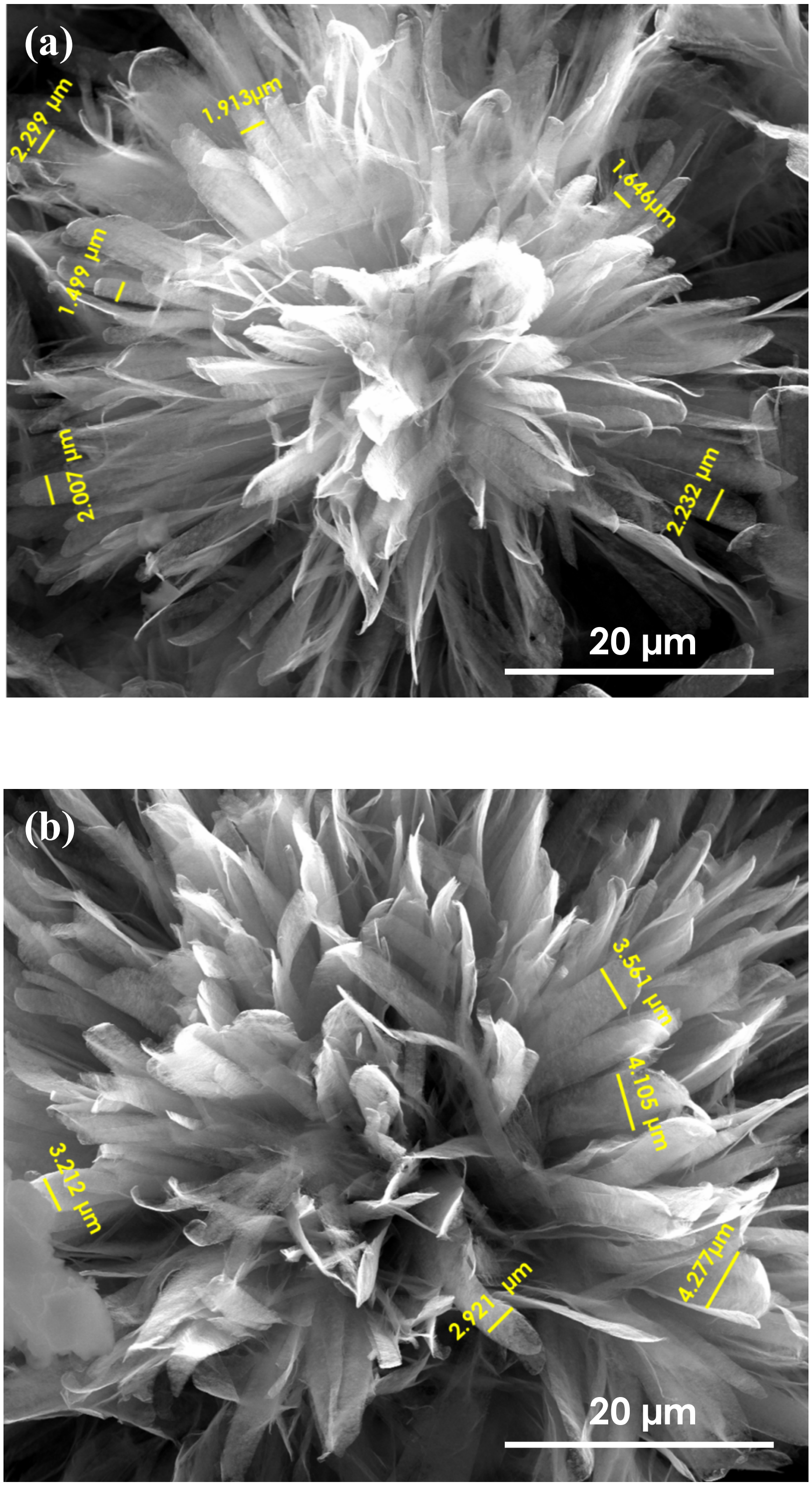

Fig. S8. SEM images of ZnO Nanoflowers showing the increase in the dimensions of petals (a) before and (b) after straining

Table S2. Summary of photocurrent densities of ZnO electrodes at 0 V_{bias} (vs. Ag/AgCl) after straining up to 2000 cycles.

ZnO thin film Morphologies	J (mA cm^{-2})					
	Flat	10-cycles	50-cycles	100-cycles	500-cycles	2000-cycles
**Nanoflower**	1.54	1.62	1.16	0.29	-	-
**Nanosheet**	1.14	0.61	0.37	-	-	-
**Nanorod**	0.87	1.04	0.57	0.57	0.45	0.41
